# Retrospective and Prospective

**Published:** 1883-12

**Authors:** 


### RETROSPECTIVE AND PROSPECTIVE.

This number closes the Fourth Volume of the INDEPENDENT PRACTITIONER. Perhaps a brief review of its past history will not here be out of place.

Founded by B. M. Wilkerson, M. D., D. D. S., its initial number was issued from Baltimore, January 1st, 1880. Its editors were Harvey L. Byrd, A. M., M. D., and Dr. Wilkerson. It was intended as a connecting link between the mother profession and her specialty offspring, and it was devoted to medical, surgical, obstetrical, dental, and hygienic science. In May, 1881, the journal was removed to New York. In September, of the same year, Dr. Byrd retired from the editorship, and Dr. Wilkerson assumed the sole editorial charge. The number for January, 1882, announced as assistant editors, Geo. H. Rohé, M. D., and Geo. W. Field, D. D. S., with a very able corps of collaborators. Drs. Rohé and Field withdrew early in the year, and Dr. Wilkerson had sole editorial charge until January, 1883, when he withdrew, because of failing health, and Leigh H. Hunt, M. D., and W. C. Barrett, M. D., D. D. S., were engaged as editors. Dr. Hunt resigned in April, and since that time Dr. Barrett has been its editor. The health of Dr. Wilkerson continued to decline, and in May it became necessary for him to relinquish the business management, of which he had so far been in charge. It was therefore essential that there should be a change of proprietors. So far, the journal had been independent of all manufacturing or mercantile interests, and so creditable had been its record that those who had felt an interest, especially in its dental department, were averse to allowing it to become the organ of any advertising firm. A conference of dentists was accordingly held in New York, and it was determined to form an association for its purchase and future publication. This was accordingly done, and the INDEPENDENT PRACTITIONER passed into the hands of its present managers. All this was not accomplished with the expectation of pecuniary profit, but that an independent journal might still be maintained that should be in spirit and in deed what its name indicated. The men who were to assume its management loved the profession to which they have devoted their lives, and here seemed to open a field in which they might unselfishly labor to advance the best interests of dentistry. They were not ignorant of the severe demands which the proper conduct of such a journal would make upon their time, but they were willing to devote themselves to what they took to be a call of duty, feeling assured that they would be loyally supported by every man who really loved his profession. They take an honest pride in saying that, so far, they have not miscalculated the prospects.

The attempt to make one periodical the exponent of both a general and special practice, to satisfactorily conduct it as both a medical and dental journal, was not in all respects successful.

When the present proprietors assumed control, the prospectus for the volume had already been published, and they have honestly striven to carry out the pledges made to its subscribers. But the fact of its being in the hands of dentists has operated against its medical department, and it has been impossible to obtain such medical articles as were desirable. We have to announce, then, that so long as this journal shall be conducted by its present proprietors, it will be exclusively devoted to dental practice, and only incidentally to medicine. We make this frank declaration, that present or intending subscribers may not be in ignorance as to its future scope.

For months the proprietors have been laboring to perfect arrangements for the coming year that shall make of this our ideal of a dental journal. We have already secured some of the ablest writers in the profession, and have engaged correspondents in all quarters of the globe, who will furnish it with all the freshest thought and news of the dental world. Already we have so much of original matter offered us that, as may be seen in this number, we are obliged to give up nearly the whole of its pages to that which will not appear elsewhere, unless the matter be borrowed by our contemporaries. With the number for January, 1884, a number of improvements in the make-up will be introduced, and we sincerely believe that the INDEPENDENT PRACTITIONER will begin a long career of usefulness and honor.

